# Toxicity of phthalate esters exposure to carp (*Cyprinus carpio*) and antioxidant response by biomarker

**DOI:** 10.1007/s10646-014-1194-x

**Published:** 2014-01-29

**Authors:** Xiaoxiang Zhao, Ying Gao, Mingliang Qi

**Affiliations:** College of Environmental Science and Engineering, Donghua University, Shanghai, 201620 People’s Republic of China

**Keywords:** Phthalate esters, Toxicity, Biomarker, Carp

## Abstract

To study the toxic effects of phthalate esters on the aquatic creatures, carps were exposed to dibutyl phthalate (DBP) and di-2-ethylhexyl phthalate (DEHP) of six different concentrations for 96 h-LC_50_ measurements. It shows that the 96 h-LC_50_ is 16.30 and 37.95 mg L^−1^, thus the safe concentration (1/10LC_50_) is 1.63 mg L^−1^. The activities of xanthine oxidase (XOD) and catalase (CAT) were measured in liver to carp exposure for single or combinations of DBP and DEHP. The quantity of malonic dialdehyde (MDA) was also measured in the same way. XOD, CAT and MDA had shown an evident change while exposure time and concentration increased, combined exposure can aggravate this change. They might be used as early warning indicators and monitors, and have potentials in the ecological risk assessment.

## Introduction

Phthalate esters (PAEs) are widely used as plasticizers in a large variety of daily products. However, they can be easily released into the water environment and are identified as one of the most prevalent organic pollutants (Hutzing 1984). The amount of PAEs, including dibutyl phthalate (DBP) and di-2-ethylhexyl phthalate (DEHP) used for the industrial production all over the world is estimated to be about 4 million tons each year (Silva et al. 2003). Due to health concerns, the European Union has issued a ban on sales of PVC infant biting toys containing PAEs on November 20, 1999 (Biedermann-Brem et al. 2008), and Japan has also made a decision to forbid using plastic gloves containing DEHP in food production since 2001 (Stellman 1998; Tanaka 2005). DBP and DEHP are commonly used as plasticizers in plastic industry (Bove and Dalven 1984), which can be exposed to human body through direct use or by indirect means after releasing into food and water, such as breathing, drinking water, diet and skin, raising public concerns.

During the enzyme process producing free radicals, xanthine oxidase (XOD) is a vital catalytic enzyme. Under its catalytic action, xanthine and hypoxanthine can be oxidized to uric acid via giving single-electron or double-electron to O_2_, simultaneously creating active oxygen radicals (Banerjee et al. 2003; Gille et al. 2001). The main physiological action of catalase (CAT) is to catalyze H_2_O_2_ to split into H_2_O and O_2_. Malonic dialdehyde (MDA) is the product of free radicals attacking unsaturated fatty acid, and can interact with the free amino of protein, inducing cell injuries due to protein intercellular and intracellular crosslink (Papadimitriou and Loumbourdis 2002). They will have special functions and close relations centered on the production and elimination of free radicals.

Currently, researches of PAEs mainly focus on the toxicity of single pollutant, but organisms are actually exposed to the mixed conditions. In this study, carps were exposed to the mixture of DBP and DEHP and the influence of single and mixed exposure on the activity of XOD and CAT, as well as the quantity of MDA were studied. Further discussion was performed on the changes of antioxidase activity and the induced oxidative damages, providing scientific basis for water standards in environmental monitoring.

## Materials and methods

### Instruments and reagents

UC-1800PC spectrophotometer (MAPADA Corp.); Hydrolab water analyser (HACH Corp.); CT14RD table model high speed centrifuge (Shanghai Tianmei Corp.); glass cuvette and quartz cuvette; homogenizer machine and so on.

DBP and DEHP were of analytic grade and were bought from Traditional Chinese Medicine, China. Acetone, which was used as a cosolvent, was bought from Traditional Chinese Medicine, China.

### Experimental material

The fish used in the experiments are in accordance with “The Rules of Experimental Animal Management”, which is used for protection of experimental animal welfare and managed by the Chinese government. The fish were caught by permission of Shanghai Ministry of Environmental Protection and approved by Shanghai Ethical Committee. They were handled in such a way to minimize stress and discomfort.

Carps (*Cyprinus carpio*) are 8 ± 1 cm in body length and weighted of 30 ± 5 g, which were bought from Fangta market at Songjiang district in Shanghai. Water was aerated continuously for 24 h and the temperature and pH were monitored to ensure that the water was applicable for the experiments. The fish were first domesticated in the aquariums of the size 62 × 33 × 28 cm with illumination for 13–14 h everyday at 20 ± 2 °C for 7 days, and fed once per day. The water should to be replaced timely.

### Acute toxicity test

The experimental procedures for acute toxicity tests were followed according to the reference (Kenaga 1981). Six groups of different concentrations based on the preliminary experiments were set. Each group was assayed in duplicate. Blank controls using acetone were also prepared for the tests. The 96 h toxicity tests were performed with no feeding in each aquarium containing 30 fish and 30 L water. The water was replaced by half at a fixed time every day. The toxic effects of fish should to be observed closely and the dead fish should be removed timely. Then the mortality rates can be calculated. The evaluation methods of safe concentration can be found in the reference (Verm et al. 1984).

### Chronic toxicity test

On the basis of acute toxicity tests, the concentrations of DBP were set up at three levels: 1.63 mg L^−1^ (1/10LC_50_), 3.26 mg L^−1^ (1/5LC_50_) and 8.15 mg L^−1^ (1/2LC_50_). The concentration of DEHP was 3.80 mg L^−1^ (1/10LC_50_), 7.59 mg L^−1^ (1/5LC_50_) and 18.98 mg L^−1^ (1/2LC_50_). The concentration of mixed exposure was 1/20LC_50_ (DBP + DEHP), 1/10LC_50_ (DBP + DEHP) and 1/4LC_50_ (DBP + DEHP). Blank controls were also prepared. The water was replaced once per day and the fish were fed every other day. The residual food and excrete were removed by siphon. The activity of enzyme was determined with an amount of 10 fish in the 1st, 3rd, 5th, 7th and 9th day after the carps were exposed.

In each experiment, 10 fish were collected randomly from different groups individually, which were dissected to get the livers after exsanguinations. The whole process was operated on the ice. The livers were washed with a 0.86 % saline solution at 4 °C, dried and weighed, and put into the homogenizer immediately with Tris–HCl buffer solution at pH 8.0 using a mass ratio of 1:9. The serous fluid was collected in centrifuge tubes and centrifuged at 4,500 r min^−1^ for 15 min. The supernatant was collected for determining the quantity of XOD, CAT, MDA and protein.

### Measuring standards and methods

The quantity of protein was determined by Lowry Method (Lowry et al. 1951), using bovine serum albumin (BSA) as a standard protein. The activity of XOD, CAT and the MDA quantity were determined by ELISA (bought from Nanjing Jiancheng Bioengineering Institute), and was accomplished according to the protocol provided by the manufacturer. The enzymatic activity unit of XOD was defined as the amount of enzyme consumed for transformation of 1 μmol substrate with 1 g protein at 37 °C. Likely, the enzymatic activity unit of CAT was defined as the amount of H_2_O_2_.

### Data analysis

All the data were statistically processed by Excel and expressed by the average number and standard deviation of triplicate results, and *t* tests were carried out by software Origin 8.0. To adopt the probability unit-concentration logarithmic method to calculate the carp 96 h half lethal concentration (96 h-LC_50_) of DEHP and DBP, and conduct tabulation analysis.

## Results

### Acute toxicity test

In the acute toxicity test, carps in each group showed different intoxication symptoms after the exposure for 6 h. In the group of higher concentration, carps were fully aroused shortly after contacting the solution. Their opercula moved fast, along with cylinder attacking. After 2 h, some carps appeared to be anoxic and lost their balance, and sometimes shook the whole body. After 4 h, carps moved slowly and appeared to get together at the bottom of the aquariums. After 8 h, some dead fish emerged. By contrast, the symptoms appeared in the low concentration groups were later, but the intoxication features were the same as that in high concentration groups. The dead fish were found after exposure for 28 h.

The results of the 96 h-LC_50_ acute toxicity test of DBP and DEHP are shown in Table 1. According to the table, the mortality of carp was increased with the increase of DBP concentration within 96 h and their linearity was remarkable. Therefore, the 96 h-LC_50_ of 16.30 mg L^−1^ for DBP to carp can be obtained using the probability unit-density logarithm method, thus the safe concentration (1/10LC_50_) was 1.63 mg L^−1^. Likely, the 96 h-LC_50_ of DEHP to carp was 37.95 mg L^−1^, thus the safe concentration was 3.80 mg L^−1^.

**Table 1:** 96 h-LC_50_ of DBP and DEHP to carp

Compound	LC_50_ (mg L^−1^)	95 % confidence (mg L^−1^)	Regression equation	Correlation coefficient (R^2^)
DBP	16.30	16.21–16.39	y = 5.725x − 1.940	0.991
DEHP	37.95	37.87–38.03	y = 6.688x − 5.562	0.974

### Influence of DBP or DEHP single exposure on XOD activity in liver

**Fig. 1:**
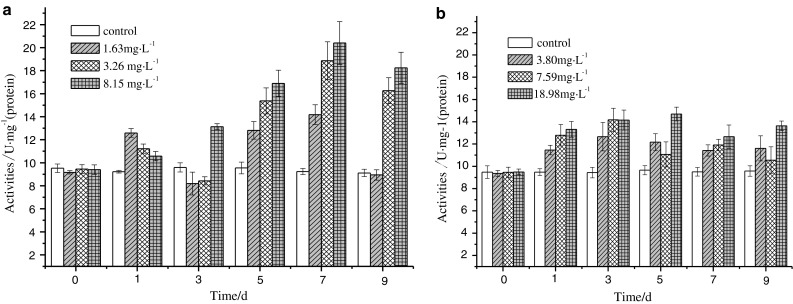
DBP (**a**) and DEHP (**b**) influence on XOD activity

As can be seen from Table 1, livers from groups exposed to DBP or DEHP were both induced significantly. From Fig. 1a, the activities of XOD in all the groups were much higher than that of the control group (*p* < 0.01) in the first day of exposure, subsequently increased with the increasing of exposure time. The XOD activities in all the groups reached a maximum in the 7th day and were 14.18, 18.86, 20.40 U mg^−1^ (protein), respectively. From Fig. 1a, the XOD was increased with the increase of the exposure time, indicating the dosage effect and time effect connections.

From Fig. 1b, the activities of XOD in all the groups were increased significantly as compared with the control group (*p* < 0.01) in the first day of exposure, indicating a ratio of 1.22, 1.35 and 1.40, respectively. The XOD activities in groups of concentrations of 3.80 and 7.59 mg L^−1^ both reached a maximum in the 3rd day, at 12.66 and 14.17 U mg^−1^ (protein), respectively. For the group of the concentration of 18.98 mg L^−1^, the XOD activity reached a maximum of 14.69 U mg^−1^ (protein) in the 5th day. From Fig. 1b, the XOD in all groups were induced but with no time effect connection except high concentration groups with the increase of exposure time. The XOD activities in high concentration groups were induced more obviously with a certain concentration effect connection.

It can be seen that the DBP had more significant inducement on the liver of carps than DEHP and XOD can be selected as a biomarker to evaluate the early pollution of DBP and DEHP.

### Influence of DBP or DEHP single exposure on CAT activity in liver

**Fig. 2:**
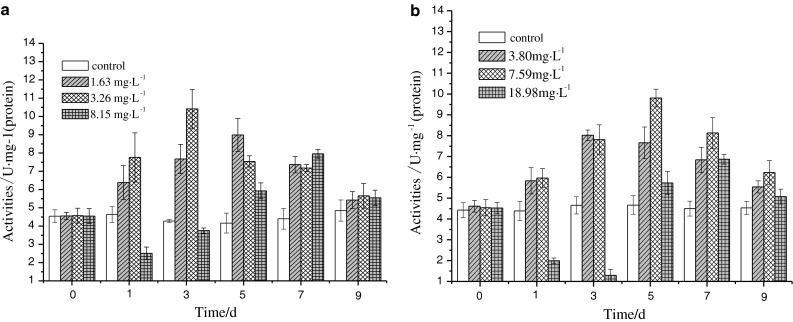
DBP (**a**) and DEHP (**b**) influence on CAT activity

From Fig. 2, the CAT activity varied significantly under the pressure of DBP and DEHP. It can be seen from Fig. 2a that the CAT activity in the groups of concentrations of 1.63 and 3.26 mg L^−1^ were induced significantly (*p* < 0.05) as compared with the control group in the first day of exposure, whereas the CAT activity in the group of concentration 8.15 mg L^−1^ was restrained remarkably (*p* < 0.01) as low as 2.51 U mg^−1^ (protein). The tendency of inducement for enzymatic activity in all groups was enhanced with the increase of exposure time. In the 5th day, the CAT activity reached a maximum at 8.99 U mg^−1^ (protein) for the group of concentration of 1.63 mg L^−1^. The CAT activity in the group of concentration of 3.26 mg L^−1^ reached a maximum at 10.41 U mg^−1^ (protein) in the 3rd day. For the group of concentration of 8.15 mg L^−1^, the CAT activity reached a maximum at 7.95 U mg^−1^ (protein) in the 7th day.

From Fig. 2b, no obvious variation of CAT activity was observed for all groups as compared with control group in the first day of exposure. However, the CAT activities in all the groups were gradually induced with increase of exposure time, and reached maximums at 6.35 U mg^−1^ (protein) in the 3rd day in the group of concentration of 3.80 mg L^−1^, 6.47 U mg^−1^ (protein) in the 5th day in the group of concentration 3.26 mg L^−1^ and 6.20 U mg^−1^ (protein) in the 7th day in the group of concentration of 18.98 mg L^−1^.

### Influence of DBP or DEHP single exposure on MDA quantity in liver

**Fig. 3:**
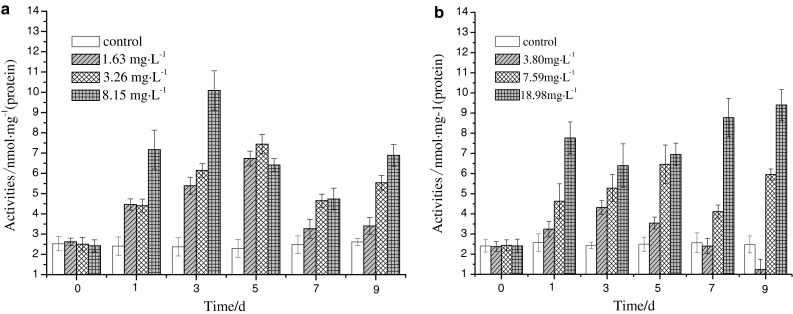
DBP (**a**) and DEHP (**b**) influence on MDA

It can be observed from Fig. 3 that the MDA quantity in the liver varied evidently under the pressure of DBP and DEHP. In the first exposure day, the MDA quantity in all groups increased significantly (*p* < 0.01), which reached maximums in the 5th day at 6.73 and 7.44 nmol mg^−1^ (protein) for groups of concentrations of 1.63 and 3.26 mg L^−1^ respectively. For the groups of concentration of 8.15 mg L^−1^, the MDA quantity reached a maximum at 10.09 nmol mg^−1^ (protein) in the 3rd day. During the exposure period, the MDA quantity in all groups was much higher than that in control groups, indicating that different concentrations of DBP had different degrees of oxidation damages to organisms.

With the increase of exposure time, the MDA quantity in the group of concentration of 3.80 mg L^−1^ initially was increased and subsequently decreased, while for groups of concentrations of 7.59 and 18.98 mg L^−1^, the MDA quantity increased continuously and reached maximums at 5.28 and 9.40 nmol mg^−1^ (protein) in the 5th and 9th day, respectively. In the last exposure day, the MDA quantity in the group of concentration of 3.80 mg L^−1^ was decreased significantly as compared with the control group (*p* < 0.05), suggesting that the organisms can eliminate the oxidation damages resulted from pollutants by adjustment with their antioxidant systems in the low dose exposure.

It was shown that the MDA quantity varied obviously under single exposure of DBP or DEHP, so the MDA can be selected as a biomarker to evaluate the early pollution of DBP and DEHP.

### Influence of DBP or DEHP single and combined exposure on XOD activity, CAT activity and MDA quantity in liver

**Fig. 4:**
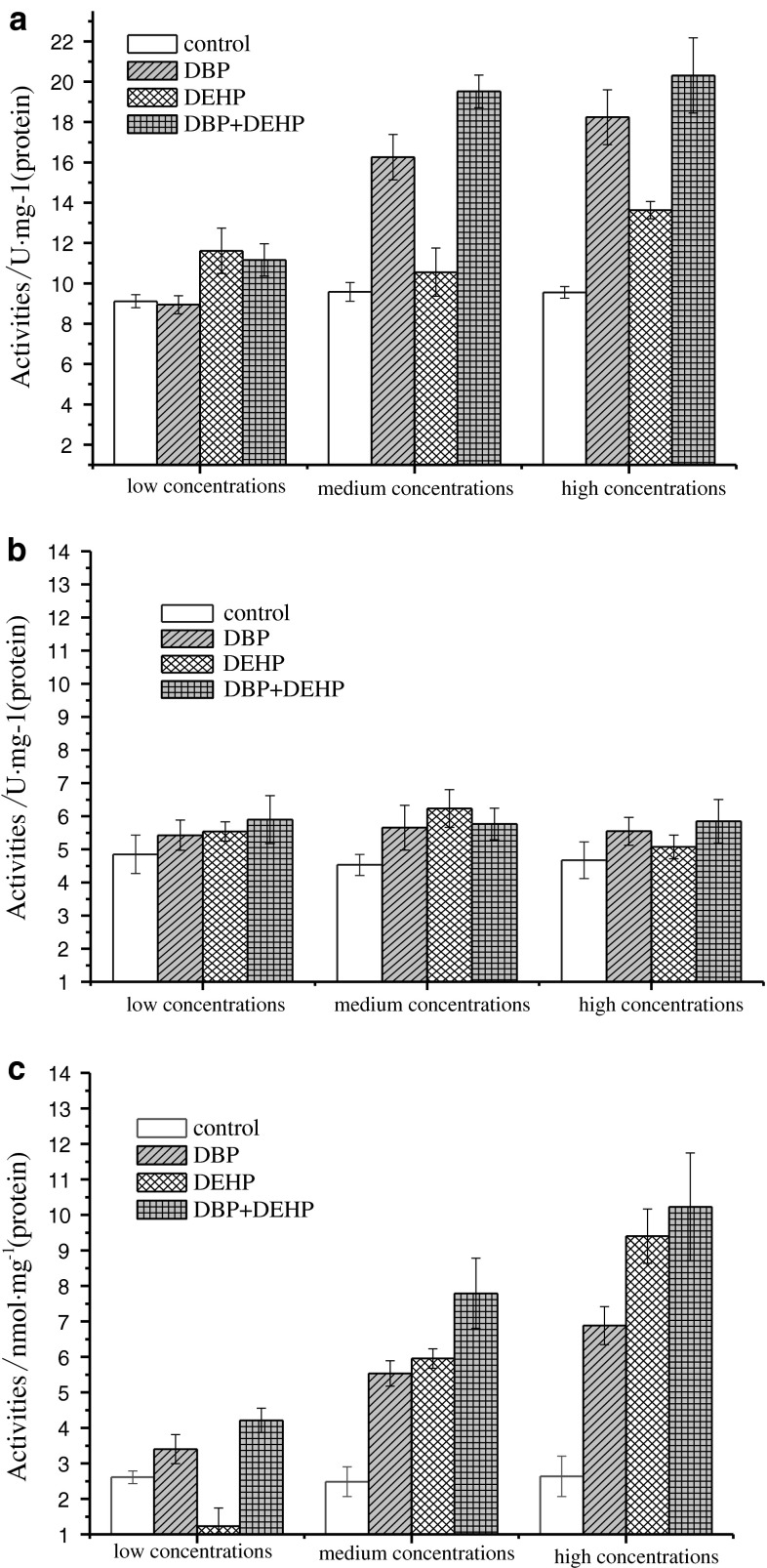
Single and combination of DBP and DEHP influence on **a** XOD activity, **b** CAT activity and **c** MDA activity

It can be seen from Fig. 4a that the degree of effects of combined exposure on XOD activity in the low-concentration groups was between single exposure to DBP and DEHP, and the XOD activity was much higher than that of the control groups (*p* < 0.01). However, in the groups at medium and high concentrations, the synergistic effects of combined exposure in contrast with single exposure on the XOD activity were observed.

It can be observed from Fig. 4b that the influence on CAT activity under combined exposure was stronger than single exposures in the low concentration groups, and the CAT activity was obviously higher than that in the control groups (*p* < 0.01). The influence on CAT activity under combined exposure was between single exposure to DBP and DEHP in the medium concentration groups, and the CAT activity was also much higher than that in the control groups (*p* < 0.01). In the high concentration groups, the influence of combined exposures was stronger than the single exposures, showing the synergistic effects.

From Fig. 4c, it can be obtained that the effects of combined exposures on the MDA quantity were stronger than the single exposure in these three concentration groups, indicating that synergistic effects were induced on the MDA quantity.

## Discussion

Xanthine oxidase is an important enzyme for the metabolism of nucleic acid in the cytolymph of animal liver and heart. It is also the main enzyme for the free radicals production in liver, heart and other organs (Fang and Li 1989). The rising of XOD quantity in liver and blood may be the main reason for liver damages, but the direct influence on active oxygen needs further investigations (Gill and Tuteja 2010). The results indicated that the toxic effects were enhanced with the increase of exposure time under single or combined pressures of DBP and DEHP. The XOD activity was constantly increased in liver, which was an important target organ of DBP and DEHP, and more free radicals tended to be produced. Under the single exposure of DBP, the XOD activity in liver was gradually increased with the increase of exposure concentration and time, somehow showing the concentration effect and time effect connections. However, under the single exposure of DEHP, although the XOD activity was induced remarkably, the time effect connection was not observed. In contrast to low concentration groups, the inducement was more significant for high-concentration groups, somehow indicating the concentration effect connection. The synergistic effects on the XOD activity were observed during the combined exposure of DBP and DEHP in the medium and high concentration groups. It can be obtained that the XOD activity in the liver of carps is sensitive to the pressure of DBP and DEHP, and can be selected as the biomarker for water monitoring.

Catalase is one of the key enzymes in the antioxidant system, which can eliminate the H_2_O_2_ produced with reactive oxygen free radicals catalyzed by superoxide dismutases, alleviating the damages to organisms (Nordberg and Arnér 2001). The results showed that the CAT activity in liver varied significantly in all groups as the exposure time increased. When the CAT activity was induced, it was beneficial to eliminate the excess H_2_O_2_ to protect the cells from oxidative damages. However, when the H_2_O_2_ elimination capacity by CAT was saturated, the excess H_2_O_2_ accumulated and the activity of CAT was restrained, then the damages to cells happened. From the results of the CAT activity in liver under DBP exposure, the CAT activity was firstly induced and then restrained in the same exposure dose but different exposure time. In the highest concentration groups, the CAT activity was restrained in the first three days. Under the same exposure time but different exposure dose, the CAT activity inducement can achieve a peak value within 9 days, indicating that the CAT activity variation can reflect the influence of early DBP exposure on carps. From the results of CAT activity in liver under DEHP exposure, the CAT activity in the two groups of low concentration appeared to be induced and then be restrained as the exposure time increased. It meant that excess active oxygen was produced in the beginning. From Fig. 2b, it can be seen that the inducement peak appeared later in the medium concentration groups so that the increase of the CAT activity protected the cells from oxidative damages. The Fig. 2b also showed that the CAT activity was firstly restrained in the highest DEHP concentration groups, and reached a minimum in the 3rd day, then increased sharply and reached a maximum in the 7th day. The possible reason is that the liver cells of carps were damaged by high concentration of DEHP, resulting in of inhibition of the CAT activity. However, it can be known from the acute toxicity tests that DEHP was not the acute toxic compound, the CAT activity were induced again after the inhibition peak. Under the combined exposure of DBP and DEHP, there were no significant synergistic or antagonistic effects, probably because of the adjustments by the antioxidant systems in carps.

Free radicals are produced through enzymatic and non-enzymatic systems in organisms, the latter can attack the polyunsaturated fatty (PUFA) in cell membranes, leading to lipid peroxidation and formation of lipid peroxide. Oxygen free radicals not only can cause cell damages by over oxidation of PUFA, but also can cause badly damages to membrane structure by breaking down the lipid peroxide (Dizdaroglu 2002). Thus, the MDA quantity can reflect the level of lipid peroxidation and indirectly reflect the level of attacks by free radicals. The results showed that the lipid peroxidation level increased as the DBP exposure concentration increased. The MDA quantities in all groups were detected to be increased notably after the first exposure day (*p* < 0.01) and were much higher than the blank group though there were fluctuations later, indicating that oxidative damages to carps were resulted from the DBP exposure. The molecular mechanism is probably that DBP restrained the activity of antioxidant enzymes, resulting in the accumulation of free radicals (Gałażyn-Sidorczuk et al. 2009). It was also possible that the metabolism of excess DBP can produce excess free radicals, which can attack the amido or imino groups in amino acid and unsaturated fatty acids in lipid bi-layer by metal ion catalyzing oxidation system. And finally, lipid peroxide such as MDA, hydroxyl and new free radicals were produced (He et al. 1999). The final MDA quantity was reduced under the low concentration exposure, showing that the oxidative damages can be eliminated by its antioxidant systems. However, the variation tendency of MDA quantity in medium and high concentration groups was similar to that of the DBP exposure groups. Under the combined exposure of DBP and DEHP, the MDA quantities in all groups were higher than single exposures indicating that worse oxidative damages were induced.

Due to the low concentration of phthalate esters and the complex analytical background, we have not measured the actual concentration of DHP and DEHP in the exposure medium or the organism samples. Nevertheless, strong relevance between the antioxidant enzyme activity in liver of carps and the pollutant dose still could be found in our study. Thus, we suggest that the antioxidant enzyme index may be used as the toxicological indicators of early plasticizers pollution for aquatic organisms monitoring.

## Conclusions

1. The half lethal concentrations (96 h-LC_50_) of DBP and DEHP were 16.30 and 37.95 mg L^−1^, respectively, belonging to the high level toxic compounds.
2. Liver is an important target organ. The XOD activity, CAT activity and MDA quantity varied notably as the exposure time and concentration increased and the combined exposure can aggravate this change. It could be suggested that XOD, CAT and MDA are a series of effective biomarkers to indicate the early pollution from DBP and DEHP.
3. The influence of DBP and EDHP on antioxidant enzyme in liver of carps was a dynamic process, which is in accordance with the toxicological rules. According to the typical impacts of plasticizers on aquatic ecosystem nowadays, we suggest the antioxidant enzyme index may be used as the toxicological indicators for aquatic organisms monitoring.
